# Long-term aerobic exercise and omega-3 supplementation modulate osteoporosis through inflammatory mechanisms in post-menopausal women: a randomized, repeated measures study

**DOI:** 10.1186/1743-7075-8-71

**Published:** 2011-10-15

**Authors:** Bakhtyar Tartibian, Behzad Hajizadeh Maleki, Jill Kanaley, Karim Sadeghi

**Affiliations:** 1 Department of Exercise Physiology, Faculty of Physical Education and Sport Science, Urmia University, Urmia, Iran; 2 Department of Exercise Physiology, Faculty of Physical Education and Sport Science, Urmia University, Urmia, Iran; 3 Department of Nutrition and Exercise Physiology, University of Missouri, Columbia, USA; 4 Department of English Language, Faculty of Humanities, Urmia University, Urmia, Iran

**Keywords:** Bone Mineral Density, Inflammation, Post-menopausal Women, Omega-3 Fatty Acids, Physical Activity

## Abstract

**Background:**

Evidence indicates that dietary fats and physical activity influence bone health. The purpose of this study was to examine the effects of long-term aerobic exercise and omega-3 (N-3) supplementation on serum inflammatory markers, bone mineral density (BMD), and bone biomarkers in post-menopausal women.

**Methods:**

Seventy-nine healthy sedentary post-menopausal women aged 58-78 years participated in this study. Subjects were randomized to one of 4 groups: exercise + supplement (E+S, n = 21), exercise (E, n = 20), supplement (S, n = 20), and control (Con, n = 18) groups. The subjects in the E+S and E groups performed aerobic exercise training (walking and jogging) up to 65% of HR_max_, three times a week for 24 weeks. Subjects in the E+S and S groups consumed 1000 mg/d N-3 for 24 weeks. The lumbar spine (L_2_-L_4_) and femoral neck BMD, serum tumor necrosis factor (TNF) α, interleukin (IL) 6, prostaglandin (PG) E_2_, estrogen, osteocalcin, 1, 25-dihydroxyvitamin D_3 _(1, 25 Vit D), C-telopeptide (CTX), parathyroid hormone (PTH) and calcitonin (CT) were measured at baseline, the end of week 12 and 24.

**Results:**

Serum estrogen, osteocalcin, 1, 25 Vit D, CT, L_2_-L_4 _and femoral neck BMD measures increased (*P *< 0.05) and the serum CTX, PTH, TNF-α, IL-6, and PGE_2 _decreased (*P *< 0.05) in E + S group after the 24 wk intervention but not in the E or S intervention groups. L_2_-L_4 _and femoral neck BMD, estrogen, osteocalcin, and CT were negatively (*P *< 0.05) correlated with TNF-α and PGE_2_. PTH and CT were correlated positively and negatively with IL-6, respectively (*P *< 0.05).

**Conclusions:**

The present study demonstrates that long-term aerobic exercise training plus N-3 supplementation have a synergistic effect in attenuating inflammation and augmenting BMD in post-menopausal osteoporosis.

## Background

With an aging society, osteoporosis has a profound economic impact [1], and primarily affects postmenopausal women. The estrogen deficiency accompanying menopause induces bone loss [2], with an abrupt decline after menopause. Prophylactic interventions to suppress this decline in bone mineral density (BMD) after menopause are needed [1].

Several lines of evidence indicate that inflammation may contribute to the disorder of osteoporosis [3-5]. Among older women, higher concentrations of inflammatory markers have been associated with an increased risk of incident fracture [6]. Pro-inflammatory cytokines, which are critical mediators of inflammatory responses, have also been found to regulate bone metabolism, even in individuals without immunological diseases [7]. Interleukin (IL) 6, has been shown to promote osteoclast differentiation and activation [8], while tumor necrosis factor (TNF) α, has been shown to stimulate bone resorption and to inhibit bone formation [9]. Prostaglandins (PGs), especially PGE_2_, are also known to be potent activators of bone remodeling [10,11].

Evidence indicates that dietary fats can also influence bone health [12]. In particular, the omega-3 (N-3) polyunsaturated fatty acids (PUFAs) may be beneficial, as they have been shown to inhibit osteoclast activity and enhance osteoblast activity in animals [13]. Optimal quantities of N-3 PUFAs, thus, appear to inhibit bone resorption and promote bone formation. One study has shown that twelve months of eicosapentaenoic acid (EPA) supplementation increases BMD in post-menopausal women [14]. Beneficial effects of N-3 PUFAs on markers of bone resorption and formation [15-17], as well as on inflammatory markers such as TNF-α, IL-1β, IL-6, and PGE_2 _in animal and human studies [18], have also been reported. Our laboratory recently investigated the effects of ingestion of N-3 PUFA and aerobic exercise intervention on the calcium regulating hormones but not on BMD in healthy post-menopausal women. These results indicated that consuming 1000 mg·day^-1 ^of N-3 PUFA during 16 weeks of moderate intensity weight-bearing exercise training significantly increased CT and estrogen levels and decreased PTH levels [12].

In addition, physical activity plays an important role in the prevention of osteoporosis via development as well as maintenance of BMD [2,19]. It is commonly accepted that participation in exercise provides an osteogenic stimulus to the bones [20], by increasing serum concentrations of bone formation markers [12,21-26] as well as decreasing bone resorption markers [12,21,25-30]. Likewise moderate intensity aerobic exercise has also been shown to attenuate the serum markers of inflammation in post-menopausal and older women [31]. Recently a 10 month randomized trial aerobic exercise training showed reduced C-reactive protein (CRP), IL-6, and IL-18 concentrations among older men and women [32]. These changes in inflammatory indices may contribute significantly to the inverse relationship between physical activity and osteoporosis.

Considering the well-known anti-inflammatory effects of exercise training [31-33], N-3 PUFAs [18], and the strong association between inflammatory markers and menopause-related osteoporosis [3-6], we hypothesized that the exercise training as well as N-3 PUFA supplementation would be effective in reducing chronic inflammation in post-menopausal women, and those changes in circulating inflammatory markers would be related to improvements in osteoporosis control. Thus, the purposes of this study were to determine whether 1) moderate intensity aerobic exercise training plus N-3 PUFA supplementation are effective in reducing serum concentrations of inflammatory markers IL-6, TNF-α, and PGE_2_, 2) moderate intensity aerobic exercise training plus N-3 PUFA supplementation are effective at improving BMD, and suppressing bone resorption; and 3) changes in inflammatory markers are associated with changes in BMD biomarkers following 24 weeks of aerobic exercise and N-3 PUFA supplementation in post-menopausal women.

## Materials and methods

### Experimental Design and Subjects

Seventy-nine post-menopausal women (aged 58-78) volunteered and gave written consent for this study which was approved by the Human Studies Committee of Urmia University, IRAN. Subjects had bone density (lumbar spine (L_2_-L_4_) and femoral neck BMD), inflammatory markers (TNF-α, IL-6, PGE_2_), hormone concentrations (estrogen, osteocalcin, 1, 25 Vit D, CTX, PTH, CT), blood ions (Ca^2+^, phosphorus, and fatty acid composition of neutrophil extracts) measured at baseline, the end of week 12 (after 24 hours of recovery), and the end of week 24 (after 24 hours of recovery).

The women were sedentary, in good health, at least 8 years past-menopause, and taking no medications. Preliminary screening included a medical history, physical examination, and a Bruce treadmill test [34]. Subjects were randomly assigned to one of 4 groups: exercise + supplement (E+S, n = 21), exercise only (E, n = 20), supplement only (S, n = 20), and control (Con, n = 18) groups. Leisure, household, and occupational activity was estimated with the Physical Activity Scale for the Elderly Questionnaire [35] (Table 1).

**Table 1:** Individual characteristics of post-menopausal women

*Variables *Groups	**E + S **(n = 21)	**E **(n = 20)	**S **(n = 20)	**Con **(n = 18)	P <
**Age **(*yr*)	59.7 ± 2.3	61.4 ± 6.9	63.1 ± 7.5	58.9 ± 8.1	0.604
**Height **(*m*)	1.64 ± 0.14	1.67 ± 0.08	1.69 ± 0.09	1.68 ± 0.16	.6410
**Weight **(*kg*)	75.2 ± 12.2	77.5 ± 10.4	78.3 ± 16.1	75.9 ± 17.2	.8150
**BMI **(*kg·m^-2^*)	26.3 ± 4.8	25.1 ± 7.1	27.9 ± 5.4	28.5 ± 3.7	.7700
**Fat **(%)	29.8 ± 5.8	27.9 ± 7.5	29.1 ± 7.2	28.1 ± 6.8	.8050
**VO**_**2max**_	33.7 ± 4.9	32.2 ± 3.1	31.2 ± 4.5	32.7 ± 4.4	.0660
**PAS**	135 ± 60	134 ± 51	140 ± 69	138 ± 61	.7010

E+S = Exercise + Supplement; E = Exercise; S = Supplement; Con = Control; BMI = Body mass index; VO_2max _= Maximal oxygen uptake (ml^-1^·kg^-1^·min^-1^); PAS = Physical activity score. Values are mean ± SD

### Oral omega-3

The E+S and S groups were supplemented with N-3 PUFA capsules (Viva Omega-3 fish oil, Manufactured by: Viva Pharmaceutical Inc, Richmond B.C. V6V 1K8, Canada), containing 180 mg EPA and 120 mg Docosahexaenoic acid (DHA), to supply a total of 1000 mg/day [12] of N-3 PUFAs over 24 weeks. The degree of compliance with N-3 PUFA supplements, as determined by pill counts, was 96 ± 9%. Also, the incorporation of EPA and DHA into the cell membranes of neutrophils was measured. Information was collected from each individual by trained interviewers in face to face interviews based on a structured and previously validated questionnaire that included the following: socio-demographic data; years since menopause; physical activities, including hours spent sitting, standing, walking, sports, and leisure activities; medications; smoking and drinking alcohol; and other factors that may have possible confounding effects on N-3 PUFA consumption and metabolism of bone and lipid [36].

All subjects were requested to consume their usual diet throughout the period of study. A detailed diary of all types and household measures of food and drinks consumed, including brand names, was kept for baseline, week 12 and week 24 [37]. Dietary intakes did not alter more than would be expected over the 24 weeks of the study (Table 2). Daily intakes of calcium, vitamin D, carbohydrates, lipids, saturated fatty acid, mono-unsaturated fatty acid, PUFA, proteins and total energy were calculated from the daily record by the dietitian on the basis of the fifth revision of the standard tables of food composition in Japan [36]. Information on use of medications and drugs was also obtained through standard and self-reported questionnaires in accordance to the researchers' recommendations [12].

**Table 2:** Differences in dietary intakes for the women

Daily intakes Groups	E + S	E	S	Con	P <
**Total energy **(*kcal*)					
Before	2116 ± 371	2101 ± 358	2121 ± 360	2099 ± 381	0.149
After	**† **2207 ± 354	**† **2211 ± 344	2119 ± 369	2109 ± 364	0.074
**Proteins **(*g*)					
Before	81 ± 14	77 ± 21	82 ± 17	80 ± 20	0.198
After	78 ± 19	81 ± 10	79 ± 18	82 ± 12	0.357
**Carbohydrates **(*g*)					
Before	224 ± 59	218 ± 51	230 ± 58	228 ± 65	0.091
After	230 ± 66	225 ± 55	233 ± 49	227 ± 60	0.208
**Lipids **(*g*)					
Before	74 ± 12	69 ± 24	71 ± 29	73 ± 22	0.077
After	72 ± 22	74 ± 18	68 ± 23	71 ± 12	0.201
**SFA **(*g*)					
Before	24 ± 9	23 ± 6	22 ± 5	22 ± 8	0.087
After	23 ± 5	23 ± 8	23 ± 7	22 ± 5	0.141
**MUFA **(*g*)					
Before	26 ± 7	28 ± 5	27 ± 6	29 ± 9	0.536
After	27 ± 5	28 ± 9	27 ± 8	28 ± 6	0.339
**PUFA **(*g*)					
Before	12 ± 4	11 ± 7	11 ± 6	11 ± 9	0.067
After	11 ± 9	11 ± 8	11 ± 8	11 ± 6	0.094
**Total fibers **(*g*)					
Before	18 ± 9	17 ± 8	17 ± 5	18 ± 6	0.091
After	17 ± 8	18 ± 6	17 ± 9	18 ± 8	0.082
**Calcium **(*mg*)					
Before	690.4 ± 234.7	692.3 ± 244.9	694.5 ± 227.3	687.6 ± 189.9	0.092
After	694.1 ± 212.1	692.8 ± 259.1	693.4 ± 235.2	690.2 ± 204.8	0.421
**Vitamin D **(*μg*)					
Before	9.7 ± 6.5	9.6 ± 6.3	9.8 ± 6.9	9.4 ± 5.7	0.114
After	9.6 ± 6.8	9.7 ± 6.8	9.4 ± 6.5	9.7 ± 7.1	0.067
**Vitamin K **(*μg*)					
Before	435.1 ± 211.7	431.9 ± 191.2	436.2 ± 204.7	434.5 ± 197.1	0.336
After	433.8 ± 227.2	435.1 ± 201.7	433.9 ± 211.1	436.2 ± 217.4	0.081

E+S = Exercise + Supplement; E = Exercise; S = Supplement; Con = Control; BMI = Body mass index; SFA = Saturated fatty acid; MUFA = Monounsaturated fatty acid; PUFA = Polyunsaturated fatty acid

**† **P < 0.05, significantly different from baseline values (within groups, baseline *vs*. week 24).

Values are mean ± SD

### Exercise program

In the first 12 weeks of the study, subjects in E+S and E groups walked or jogged on a treadmill 25-30 min·day^-1^, 3-4 days·week^-1^, at 45-55% of their individually determined HR_max_. As their exercise tolerance improved, the intensity and duration of exercise training in the second 12 weeks of the study were increased to 40-45 min·day^-1^, 4-6 days·week^-1^; at an intensity of 55-65% of HR_max_. Adherence to the exercise prescription was documented through the use of Polar heart rate monitors, and subjects received feedback if training intensities were either too high or low in comparison with desirable intensities. Attendance was taken at each exercise session to monitor compliance with the program. Subjects were contacted if an exercise session was missed. In those women who completed the interventions, there was > 95% compliance for attendance at the exercise sessions. Nine subjects (E + S group, n = 1; E group, n = 2; S group, n = 2; Con group, n = 4) could not complete the study protocol and were excluded from the study. All exercise sessions began between 0600 and 0800 h. The S and Con group subjects were instructed to maintain their current physical activity levels during the study [12].

### Measurements

#### Blood sampling and assays

Following a 12-hour overnight fast, blood samples were taken between 0700-0800 h and collected from an antecubital vein. Samples were taken at baseline, 12 and 24 wks. Standard procedures for blood handling and processing were followed. All samples were taken 24 h after the last exercise bout. Blood samples were analyzed for serum TNF-α, IL-6, PGE_2_, estrogen, osteocalcin, 1, 25- Vit D, CTX, PTH, CT, Ca^2+^, phosphorus, and fatty acid composition of neutrophil extracts.

Serum IL-6 and TNF-α measurements were assayed using an enzyme-linked immunosorbent assay (ELISA) kit from Biosource (Nivelles, Belgium). PGE_2 _was determined using competitive enzyme immunoassay (PGE_2 _EIA kit- Monoclonal [solid plate], Ceyman-Germany). Serum estrogen levels were detected through a chemiluminescent method (Roche Diagnostics, Indianapolis, IN, USA) using an automatic immunoanalyzer. Circulating osteocalcin was measured using a previously developed radioimmunoassay (RIA). Serum 1, 25- Vit D was measured by a radioreceptor assay. Serum CTX was assessed using Serum Cross Laps One-Step ELISA (Osteometer BioTech, Herlev, Denmark). Serum PTH levels were measured through an electrochemiluminescent method, using Elecsys Systems/Modular analytics E170 (catalog number, 11972103; Roche Diagnostics). CT was determined by radioimmunoassay (RIA) with a polyclonal antiserum directed against the carboxyterminus. The antibodies were produced in rabbits against human CT, using a calcitonin kit (CIS Bio International ORIS groups, France). Serum calcium and phosphorus levels were measured by standard automated laboratory techniques. Incorporation of EPA and DHA into the cell membranes of neutrophils was analyzed using gas chromatography using known standards [12,37].

#### Bone measurements

BMD measurements were made at the anterior-posterior lumbar spine (L_2_-L_4_) and the non-dominant proximal femur, including femoral neck, using dual-energy X-ray absorptiometry (DXA; Norland XR36; Norland, Fort Atkinson, WI, USA). The coefficients of variation (CV) for the L_2_-L_4 _and femoral neck were 1.21% and 1.54% respectively.

### Statistical analysis

Group differences were determined using a one way analysis of variance (*ANOVA*) for repeated measures, for continuous variables. If the main effects *F*-ratio was significant, differences among groups were subsequently identified using a Bonferroni *post-hoc *analysis. Partial correlation and mixed model regression coefficients were used to evaluate the association between the variables studied. The statistical software program SPSS (SPSS Co, Chicago IL, version 17) for windows was used for data analysis. All statistical tests were performed and considered significant at a *P *≤ 0.05.

## Results

Baseline characteristics of the four study groups did not show any significant differences among groups in L_2_-L_4 _and femoral neck BMD, TNF-α, IL-6, PGE_2_, estrogen, osteocalcin, 1, 25 Vit D, CTX, PTH, CT, Ca^2+^, and phosphorus (*P *> 0.05) (Table 3).

**Table 3:** Baseline, Week 12, and 24 Values of Bone Mineral Density, Inflammatory Markers, and Osteoporosis Biomarkers in Different Groups of Post-Menopausal Women

Variables Groups	S+E	E	S	Con	*P <
**L_2_-L_4 _BMD **(*g/cm^2^*)					
**Baseline**	**0.79 ± 0.12**	**0.79 ± 0.12**	**0.78 ± 0.12**	**0.79 ± 0.14**	**0.995**
**Week 12**	**0.84 ± 0.14**	**0.81 ± 0.12**	**0.79 ± 0.11**	**0.79 ± 0.13**	**0.51**
*CfB*	*0.05 ± 0.15*	*0.02 ± 0.06*	*0.01 ± 0.09*	*-0.01 ± 0.06*	
*(%)*	*3.3 (0, 15.6)*	*0.7 (-1.3, 7.5)*	*0 (-1, 10)*	*0 (0, 0)*	
**Week 24**	**0.91 ± 0.07 **^**2, 3, 4 **^**†#**	**0.84 ± 0.13 **^**1, 4**^	**0.83 ± 0.1 **^**1, 4**^	**0.73 ± 0.14 ^1, 2, 3 ^†#**	*0.001
*CfB*	*0.13 ± 0.15*	*0.05 ± 0.14*	*0.05 ± 0.13*	*-0.06 ± 0.16*	
*(%)*	*15.8 (3.3, 42.5)*	*7.6 (0, 19.9)*	*1.5 (-0.2, 18)*	*-8.5 (-23.9, 0)*	
**Femoral neck BMD ***(g/cm^2^)*					
**Baseline**	**0.68 ± 0.11**	**0.67 ± 0.10**	**0.67 ± 0.10**	**0.69 ± 0.08**	**0.981**
**Week 12**	**0.78 ± 0.17 **^**2, 3, 4 **^**†**	**0.71 ± 0.15 **^**4**^	**0.71 ± 0.09**^**1, 4**^	**0.65 ± 0.07 **^**1, 2, 3**^	*0.013
*CfB*	*0.11 ± 0.21*	*0.03 ± 0.21*	*0.03 ± 0.07*	*-0.04 ± 0.06*	
*(%)*	*17.9 (-10.6, 48.9)*	*3.6 (-12.2, 21.2)*	*1.9 (0, 6.7)*	*-2.8 (-5.9, -1.4)*	
**Week 24**	**0. 87 ± 0.17 **^**2, 3, 4 **^**†#**	**0.74 ± 0.13 **^**1, 4**^	**0.74 ± 0.15 **^**1, 4**^	**0.63 ± 0.09 **^**1, 2, 3**^	*0.001
*CfB*	*0.19 ± 0.18*	*0.07 ± 0.19*	*0.07 ± 0.13*	*-0.06 ± 0.1*	
*(%)*	*19.3 (-2.5, 56.8)*	*8 (-11.2, 22.9)*	*17 (-3.4, 25.3)*	*-7.5 (-11.4, -0.3)*	
**TNF-α **(*pg/ml*)					
**Baseline**	**79 ± 15.4**	**77.6 ± 17.1**	**76.6 ± 17.3**	**79.8 ± 8.3**	**0.943**
**Week 12**	**44.9 ± 20.3 **^**2, 3, 4**^**†**	**68.5 ± 16.9**^**1, 4**^	**61.1 ± 24.2 **^**1, 4**^**†**	**83.6 ± 8.6**^**1, 2, 3**^	*0.001
*CfB*	*-34.1 ± 24.7*	*-9.2 ± 29.3*	*-16.9 ± 33.1*	*3.8 ± 6.7*	
*(%)*	*-47 (-55.7, -33.8)*	*-22.1 (-27.2, 9.3)*	*-27.4 (-50.9, -2.3)*	*5.4 (0, 12.4)*	
**Week 24**	**19.7 ± 17.4 **^**2, 3, 4**^**†#**	**58.2 ± 28.1**^**1, 3, 4**^	**34 ± 31.8 **^**1, 2, 4**^**†#**	**88.3 ± 20.6**^**1, 2, 3**^	*0.001
*CfB*	*-59.3 ± 23.7*	*-19.4 ± 31.9*	*-42.5 ± 30.8*	*8.5 ± 17.9*	
*(%)*	*-81.7 (-85.6, -78.4)*	*-16.1 (-64.1, 8.9)*	*-76.9 (-83.5, -10)*	*8.8 (0.5, 17.1)*	
**IL-6 **(*pg/ml*)					
**Baseline**	**49.3 ± 26.5**	**50.7 ± 25.1**	**48.8 ± 27.2**	**51.6 ± 20.5**	**0.991**
**Week 12**	**40.2 ± 17.3**	**49.6 ± 30.9**	**46.4 ± 29.9**	**54.6 ± 29.2**	**0.547**
*CfB*	*-9.1 ± 14.8*	*-1.1 ± 37.8*	*-2.4 ± 31.1*	*3.1 ± 31.3*	
*(%)*	*-11.2 (-24.2, 12.2)*	*-0.4 (-49.3, 72.4)*	*0.1 (-39.6, 21.6)*	*-13.5 (-37.9, 66.3)*	
**Week 24**	**21.9 ± 10 **^**2, 3, 4**^**†#**	**43 ± 26.5**^**1, 4**^	**40.8 ± 26.6**^**1, 4**^	**63 ± 30.3 **^**1, 2, 3**^	*0.001
*CfB*	*-27.4 ± 29.7*	*-7.7 ± 48.3*	*-8 ± 24.2*	*11.4 ± 37.9*	
*(%)*	*-40.8 (-76.4, -31.4)*	*-37.8 (-70.1, 155.6)*	*-24.5 (-40.7, 3)*	*20.1 (-39.8, 120)*	
**PGE_2 _**(*pg/ml*)					
**Baseline**	**16.2 ± 4.9**	**16.6 ± 5.7**	**16.6 ± 5.9**	**16.1 ± 5.3**	**0.991**
**Week 12**	**11.2 ± 4.2 **^**3, 4**^**†**	**12.8 ± 4.9**^**3, 4**^	**15.1 ± 2.7 **^**1, 2**^	**17.6 ± 2.8 **^**1, 2**^	*0.001
*CfB*	*-5 ± 6.1*	*-3.8 ± 6.6*	*-1.6 ± 7.4*	*1.6 ± 5*	
*(%)*	*-27.5 (-53.1, 10)*	*-7.4 (-29.2, 0)*	*-7 (-35.4, 26.6)*	*3.4 (-10.3, 47.3)*	
**Week 24**	**4.6 ± 2.3 **^**2, 3, 4**^**†#**	**11.3 ± 4.1 ^1, 4 ^†**	**12.6 ± 3 ^1, 4 ^†**	**17.3 ± 2.7**^**1, 2, 3**^	*0.001
*CfB*	*-11.5 ± 5.9*	*-5.3 ± 8.4*	*-4 ± 7.4*	*1.2 ± 5.4*	
*(%)*	*-69.9 (-80.1, -65)*	*-29.7 (-58.5, -2.8)*	*-32 (-51.4, 15.4)*	*6 (-17.5, 43.2)*	
**Estrogen **(*pg/ml*)					
**Baseline**	**15 ± 7.4**	**15.6 ± 8.1**	**15.8 ± 8**	**14.8 ± 8.5**	**0.983**
**Week 12**	**24 ± 7.1 **^**2, 3, 4**^**†**	**17 ± 7.7**^**1, 4**^	**18.6 ± 6 **^**1, 4**^	**12.7 ± 7.4 **^**1, 2, 3**^	*0.001
*CfB*	*8.9 ± 11*	*1.4 ± 11.1*	*2.7 ± 10.4*	*-2.1 ± 13*	
*(%)*	*65.9 (40.7, 134.2)*	*-14.4 (-36.9, 96.7)*	*-1.7 (-26, 127.5)*	*1.4 (-45.8, 35.2)*	
**Week 24**	**33.7 ± 11.9 **^**2, 3, 4**^**†#**	**20.2 ± 7.4**^**1, 4**^	**21.4 ± 8.1**^**1, 4**^	**12.9 ± 8.5 **^**1, 2, 3**^	*0.001
*CfB*	*18.7 ± 11.6*	*4.6 ± 10.4*	*5.5 ± 10.2*	*-1.8 ± 8.8*	
*(%)*	*126.6 (50, 215)*	*52.8 (-8.8, 102.4)*	*56 (-24.2, 108)*	*0 (0, 0)*	
**Osteocalcin **(*ng/ml*)					
**Baseline**	**26.3 ± 8.7**	**25.8 ± 8.4**	**26.7 ± 8.6**	**24.4 ± 7.7**	**0.891**
**Week 12**	**34.4 ± 8.4 **^**4**^**†**	**27.2 ± 8.7**	**28.5 ± 10.1**	**23.6 ± 8.2**^**1**^	*0.016
*CfB*	*8.1 ± 12.2*	*1.4 ± 12.1*	*1.8 ± 14.7*	*-0.8 ± 11*	
*(%)*	*37.8 (-13.4, 83.4)*	*6.7 (-19.9, 55.2)*	*-2.4 (-34.7, 27.8)*	*0 (-13.5, 27.4)*	
**Week 24**	**39.2 ± 8.7 **^**2, 3, 4**^**†**	**29.8 ± 10.5**^**1**^	**28.1 ± 9.6 **^**1**^	**22.6 ± 7.3 **^**1**^	*0.001
*CfB*	*12.9 ± 14.7*	*4 ± 12.9*	*1.4 ± 15.1*	*-1.8 ± 7.6*	
*(%)*	*89.1 (9.9, 131.1)*	*4.6 (-21.7, 67)*	*15.1 (-30.9, 74.4)*	*0 (-7.7, 0)*	
**1, 25 Vit D **(*pg/ml*)					
**Baseline**	**39.9 ± 19.1**	**41.5 ± 21.9**	**39.3 ± 17.2**	**42.3 ± 20.6**	**0.974**
**Week 12**	**50 ± 19.1 †**	**44.6 ± 17.9**	**45.4 ± 19.8**	**41.4 ± 15.7**	**0.648**
*CfB*	*10.1 ± 28.7*	*3 ± 30.4*	*6.1 ± 24.7*	*-1 ± 25.1*	
*(%)*	*15.1 (-8.4, 132.5)*	*-28.3 (-39, 146.5)*	*32.7 (-33.6, 103.2)*	*-11.7 (-34.7, 84.5)*	
**Week 24**	**66.9 ± 14.3 **^**2, 3, 4**^**†#**	**54.9 ± 23.1**^**1, 4**^	**49.4 ± 19.1 **^**1, 4**^	**38 ± 19.9 **^**1, 2, 3**^	*0.007
*CfB*	*26 ± 23*	*13.4 ± 26.1*	*7.2 ± 28.5*	*-4.3 ± 32.8*	
*(%)*	*62.4 (4, 204.6)*	*53 (6.8, 76.7)*	*44.9 (-34.7, 103.2)*	*-9.2 (-53.5, 62.1)*	
**CTX **(*ng/ml*)					
**Baseline**	**0.5 ± 0.2**	**0.5 ± 0.1**	**0.5 ± 0.1**	**0.5 ± 0.1**	**0.988**
**Week 12**	**0.4 ± 0.1**	**0.5 ± 0.2**	**0.5 ± 0.1**	**0.5 ± 0.1**	**0.635**
*CfB*	*-0.1 ± 0.2*	*0 ± 0.2*	*0 ± 0.2*	*0 ± 0.2*	
*(%)*	*-6.3 (-18.8, 24)*	*-21.7 (-44.2, 47.7)*	*0 (-25.3, 20.1)*	*-3.7 (-27.5, 42.8)*	
**Week 24**	**0.3 ± 0.1 **^**4**^**†**	**0.4 ± 0.1**	**0.5 ± 0.2**	**0.5 ± 0.1**^**1**^	*0.006
*CfB*	*-0.2 ± 0.2*	*-0.1 ± 0.2*	*0 ± 0.2*	*0 ± 0.2*	
*(%)*	*-32.8 (-50.2, 0.6)*	*-8.1 (-27.5, 26.3)*	*5 (-22.6, 23.2)*	*-0.9 (-25.5, 53.1)*	
**PTH **(*pg/ml*)					
**Baseline**	**90.7 ± 39.5**	**92.5 ± 46.6**	**88.1 ± 42.8**	**94.9 ± 46.6**	**0.98**
**Week 12**	**53.9 ± 34.4 **^**2, 3, 4**^**†**	**83 ± 40.5**^**1, 4**^	**87.8 ± 39.8 **^**1, 4**^	**92.9 ± 44.6 **^**1, 2, 3**^	*0.047
*CfB*	*-36.8 ± 60.3*	*-9.4 ± 58.7*	*-0.1 ± 63.6*	*-2 ± 57.2*	
*(%)*	*-57.6 (-70.4, -8.9)*	*-34.8 (-54.6, 88.6)*	*31.4 (-52.4, 63.7)*	*16.6 (-43.8, 79.2)*	
**Week 24**	**35.8 ± 19.1 **^**2, 3, 4**^**†#**	**68.8 ± 36.9**^**1, 3, 4**^	**80.8 ± 40 **^**1, 2, 4**^	**97.5 ± 40.5 **^**1, 2, 3**^	*0.001
*CfB*	*-54.9 ± 42*	*-23.6 ± 54.5*	*-7.3 ± 41.3*	*2.6 ± 66.9*	
*(%)*	*-52.4 (-67.8, -28.5)*	*-29.6 (-54.4, 19)*	*-8.9 (-31, 53.9)*	*9.9 (-41.6, 125.8)*	
**CT**(*pg/ml*)					
**Baseline**	**1.6 ± 0.4**	**1.5 ± 0.4**	**1.6 ± 0.3**	**1.6 ± 0.4**	**0.862**
**Week 12**	**12.4 ± 4.7 **^**2, 3, 4**^**†**	**7.9 ± 4.2 ^1, 4 ^†**	**6.8 ± 2.7 ^1, 4 ^†**	**1.6 ± 0.3**^**1, 2, 3**^	*0.001
*CfB*	*10.8 ± 4.8*	*6.4 ± 4.1*	*5.2 ± 2.7*	*0 ± 0.5*	
*(%)*	*709.5 (557.6, 955.2)*	*729.3 (426.1, 1114.3)*	*313.4 (182.2, 474.5)*	*6.8 (-16.3, 25.2)*	
**Week 24**	**24 ± 11.8 **^**2, 3, 4 **^**†#**	**10.2 ± 7**^**1,4**^**†**	**8.9 ± 3.4 **^**1, 4**^**†**	**1.6 ± 0.4 **^**1, 2, 3**^	*0.001
*CfB*	*22.5 ± 11.8*	*8.6 ± 6.7*	*7.2 ± 3.4*	*0 ± 0.6*	
*(%)*	*1181.8 (939.1, 2014.6)*	*838.8 (658, 1217.1)*	*435.7 (287.1, 546.7)*	*1.6 (-26.5, 42.7)*	
**Ca^2+ ^**(*mg/dl*)					
**Baseline**	**9.6 ± 0.7**	**9.5 ± 0.7**	**9.3 ± 0.7**	**9.3 ± 0.7**	**0.503**
**Week 12**	**9.3 ± 0.9**	**9.4 ± 1**	**9.3 ± 0.8**	**9.4 ± 0.7**	**0.963**
*CfB*	*-0.4 ± 1*	*-0.2 ± 1.2*	*0 ± 1.2*	*0.1 ± 1.2*	
*(%)*	*-3 (-12.4, 4.2)*	*-0.7 (-15.4, 9.2)*	*0.1 (-7.4, 2.1)*	*1.3 (-9.4, 15.6)*	
**Week 24**	**9 ± 0.8**	**9.1 ± 0.8**	**9.3 ± 0.7**	**9.4 ± 0.7**	**0.483**
*CfB*	*-0.7 ± 1.1*	*-0.5 ± 0.8*	*-0.1 ± 1.1*	*0.1 ± 1.1*	
*(%)*	*-9.7 (-12.1, 0.3)*	*-7 (-9.5, -3.1)*	*-3.9 (-9.4, 11.2)*	*4.2 (-10.1, 10.3)*	
**Phosphorus **(*mg/dl*)					
**Baseline**	**3.7 ± 0.6**	**3.8 ± 0.5**	**3.9 ± 0.7**	**3.6 ± 0.6**	**0.489**
**Week 12**	**3.6 ± 0.6**	**3.9 ± 0.6**	**3.6 ± 0.6**	**3.7 ± 0.7**	**0.608**
*CfB*	*-0.1 ± 1*	*0.1 ± 0.8*	*-0.3 ± 0.9*	*0.2 ± 0.7*	
*(%)*	*-4.2 (-16.3, 26.3)*	*3.6 (-15.7, 22.2)*	*-7.5 (-20.7, 9.8)*	*0 (-2.6, 24.1)*	
**Week 24**	**3.2 ± 0.6**	**3.5 ± 0.6**	**3.7 ± 0.8**	**3.6 ± 0.7**	**0.185**
*CfB*	*-0.5 ± 0.9*	*-0.3 ± 1*	*-0.2 ± 1.2*	*0.1 ± 0.7*	
*(%)*	*-10.7 (-35.7, 7.6)*	*-15.7 (-23.7, 2.9)*	*5 (-29, 14.8)*	*0 (-11.7, 14.1)*	

E+S = Exercise + Supplement; E = Exercise; S = Supplement; Con = Control; CfB: Change from Baseline.

*: P < 0.05, significant difference between groups.

**†**: P < 0.05, significantly different from baseline values (within groups, baseline *vs*. week 12).

**#**: P < 0.05, significantly different from week 12 values (within groups, week 12 *vs*. week 24).

Superscripts denote significant differences among the groups (E+S = 1; E = 2; S = 3; and Con = 4).

### Bone measurements

At 12 wks of the intervention, L_2_-L_4 _BMD was not significantly different than baseline values and there were no difference between group values (Table 3). However by 24 wks, the E+S intervention resulted in a significant increase in L_2_-L_4 _BMD from the baseline value (0.17 ± 0.15 g/cm^2 ^increase, *P *< 0.05). Although there were slight increases in L_2_-L_4 _BMD in the E and S groups, it was not significant and these changes were smaller (*P *< 0.05) than seen in the E+S group, but greater than seen in the control group (*P *< 0.05). Likewise the femoral neck BMD also increased in response to the E+S intervention (*P *< 0.05), with a significant increase at 12 wks as well as at 24 wks, and femoral neck BMD at 12 and 24 wk was greater than found in the other 3 groups (*P *< 0.05). The other groups showed no significant improvement in femoral neck BMD with the intervention, but higher BMD levels were seen in the E and S groups compared to the control group (*P *< 0.05). The change seen in both the L_2_-L_4 _BMD and femoral neck BMD reveals that exercise training and supplementation had a synergistic effect on the BMD.

### Inflammatory markers

Attenuated levels of TNF-α compared to baseline were observed at weeks 12 and 24 in both the E+S and the S groups (*P *< 0.05) such that they decreased from baseline by 34.1 ± 24.7 and 16.9 ± 33.1 pg.ml^-1^, respectively (Table 3). The decrease was significantly greater in the E+S group (*P *< 0.05), and both groups had a greater decrease than the E and Con group (*P *< 0.05). The exercise and N-3 PUFA supplement acted synergistically to cause the decrease in TNF-α but it appears that the supplement contributed more so to this decrease.

IL-6 levels did not change at 12 weeks, but a significant decrease was observed in the E+S group at 24 wks (Table 3). The IL-6 levels were lower in the E+S group than any of the other groups, and E and S groups were lower than the control group (*P *< 0.05). PGE_2 _decreased at both 12 and 24 wk in the E+S group, but not in the other groups. This decrease was only significantly greater than the S and Con group at 12 wk. At 12 wks, the IL-6 levels were similar between the E+S and E groups, and both groups were higher than the S and Con group (*P *< 0.05). However at 24 wk, all intervention groups showed a decrease in PGE_2 _levels from baseline, and all had greater decreases than the Con group (*P *< 0.05). The decrease seen in the E+S group was greater than in either the E (~30%) and S groups (~32%) (*P *< 0.05), and they were not different from each other.

### Hormones

Estrogen levels increased from baseline at both 12 and 24 wks in the E+S group but not in any of the other groups (*P *< 0.05) (Table 3). Further the E+S group had higher estrogen levels at these 2 time points than any of the other groups (*P *< 0.05). Calcitonin levels demonstrated a significant increase from baseline at 12 and 24 wks in all intervention groups (*P *< 0.05), and the E+S group had a greater change from baseline than the other 2 intervention groups. At 24 wks all groups still had elevated levels of CT over baseline (*P *< 0.05), but only the E+S group continued to show a significant increase again over the 12 wk measurement (*P *< 0.05). This resulted in the E+S group having greater CT levels (24.0 ± 11.8) than either of the E (10.2 ± 7.0) and S (8.9 ± 3.4) group (*P *< 0.05). The Con group (1.6 ± 0.4) demonstrated no change in the 24 wks.

Concomitantly, PTH showed a decrease in the E+S group only both at 12 and 24 weeks, while both the E and S groups showed a non-significant decline. Again the PTH levels were lower than in either of those intervention groups, which showed no difference from each other but were lower than the PTH levels in the Con group (*P *< 0.05).

At 12 and 24 weeks, osteocalcin and 1, 25 Vit D levels had increased from baseline (*P *< 0.05) in the E+S group and these levels were greater than observed in any of the other groups (*P *< 0.05). There were no differences in osteocalcin levels between the other groups, while 1, 25 Vit D was greater in the E and S group than in the control group (*P *< 0.05). C-telopepide levels decreased from baseline only at 24 wks in the E+S group, with no changes in the other groups. The E+S concentrations of CTX were only significantly greater than the Con group (*P *< 0.05).

### Serum ions

Calcium and phosphorus concentration remained stable through the 24 week intervention in all groups (Table 3). There were no differences in these levels between and of these either before or during the intervention.

### Correlations

The association between the inflammatory markers and biomarkers of BMD in all groups are shown in Table 4. Significant negative correlations were observed between L_2_-L_4 _and femoral neck BMD, estrogen, osteocalcin, and CT with inflammatory markers TNF-α (*r *= -0.289; *P *= 0.002, *r *= -0.238; *P *= 0.049, *r *= -0.283; *P *= 0.001, *r *= -0.281; *P *= 0.001, and *r *= -0.484; *P *= 0.001, respectively) and PGE_2 _(*r *= -0.219; *P *= 0.005, *r *= -0.264; *P *= 0.030, *r *= -0.223; *P *= 0.004, *r *= -0.249; *P *= 0.001, and *r *= -0.424; *P *= 0.001, respectively) (Table 4). Results from the mixed model regression showed that for each unit (pg/ml) decrease in TNF-α concentration there was an increase of 49.8 g/cm^2^, 25.9 g/cm^2^, 0.7 pg/ml, 0.7 ng/ml, and 1.7 pg/ml, respectively, in L_2_-L_4 _and femoral neck BMD, estrogen, osteocalcin, and CT. Additionally each unit (pg/ml) decrease in PGE_2 _concentrations resulted in 8.7 g/cm^2^, 6.6 g/cm^2^, 0.12 pg/ml, 0.13 ng/ml, and 0.29 pg/ml increase, respectively, in L_2_-L_4 _and femoral neck BMD, estrogen, osteocalcin, and CT in these women. However, no significant correlation was observed between 1, 25 Vit D, CTX, PTH, Ca^2+^, and phosphorus with either TNF-α or PGE_2 _(Table 4). PTH and IL-6 levels were positively correlated (*r *= 0.216; *P *= 0.005), while a negative correlation between CT and IL-6 concentrations (*r *= -0.169; *P *= 0.027) was noted. The mixed model regression showed that each unit decrease in IL-6 concentration resulted in 0.13 unit decrease in PTH levels, and each unit decrease in IL-6 concentration resulted in 0.59 unit increase in CT levels. PTH and TNF-α and PGE_2 _levels were not associated (Table 4)_._

**Table 4:** Correlation of Inflammatory Markers with Bone Mineral Density and Osteoporosis Biomarkers in Healthy Post-menopausal Women

	TNF-α (*pg/ml*)			IL-6 (*pg/ml*)			PGE_2 _(*pg/ml*)		
	
		Regression			Regression			Regression	
						
	Correlation	RC	*P <	Correlation	RC	*P <	Correlation	RC	*P <
**L_2_-L_4 _BMD **(*g/cm^2^*)	-.289	-49.8	*0.002	-.126	-25.1	0.123	-.219	-8.7	*0.005
**Femoral neck BMD **(*g/cm^2^*)	-0.238	-25.9	*0.049	-.097	-21.3	0.174	-.264	-6.6	*0.030
**Estrogen **(*pg/ml*)	-.283	-0.7	*0.001	.065	0.30	0.171	-.223	-0.12	*0.004
**Osteocalcin **(*ng/ml*)	-.281	-0.7	*0.001	-.157	-0.37	0.085	-.249	-0.13	*0.001
**1, 25-dihydroxyvitamin D_3 _**(*pg/ml*)	-0.101	-0.1	0.147	-.048	-0.06	0.545	-.123	-0.03	0.103
**CTX **(*ng/ml*)	0.143	24.1	0.078	.119	20.9	0.137	.067	2.3	0.405
**PTH **(*pg/ml*)	0.109	0.06	0.207	.216	0.13	*0.005	.115	0.01	0.162
**CT**(*pg/ml*)	-.484	-1.7	*0.001	-.169	-0.59	*0.027	-.424	-0.29	*0.001
**Ca^2+ ^**(*mg/dl*)	0.115	3.3	0.117	.007	0.48	0.850	.113	0.69	0.258
**Phosphorus **(*mg/dl*)	0.067	2.9	0.342	.036	1.68	0.596	.172	1.34	0.127

*: P < 0.05, Adjusted for the group through the study based on mixed model.

RC: Regression coefficient

### Fatty acid composition

Neutrophil phospholipids PUFA content is expressed as a percentage of total phospholipid. Fatty acid content is presented in Table 5. No significant changes (*P *> 0.05) were observed in neutrophil membrane content when comparing linoleic acid (LA), Arachidonic Acid (AA), EPA and DHA values in the E and Con groups before and after the study. Following N-3 PUFA supplementation, the neutrophil phospholipids content of DHA and EPA increased significantly (*P *< 0.05) in E+S and S groups, while the neutrophil phospholipid content of LA and AA was significantly reduced (*P *< 0.05) (Table 5).

**Table 5:** Fatty acid composition of neutrophil extracts expressed as a percentage of total fatty acid content before and after dietary supplementation

Variables Groups	E+S	E	S	Con	P <
**18:2§LA**					
Before	16.7 ± 2.8	17.1 ± 2.4	16.9 ± 3.4	17.2 ± 2.9	0.098
After	**† **6.9 ± 3.5 ^2, 4^	17.2 ± 2.7 ^1, 3^	**† **7.2 ± 3.8 ^2, 4^	16.9 ± 3.1 ^1, 3^	* 0.001
**20:4§AA**					
Before	21.7 ± 6.1	22.2 ± 3.8	22.2 ± 4.3	22.4 ± 5.1	0.121
After	**† **12.1 ± 4.2 ^2, 4^	22.7 ± 5.9 ^1, 3^	**† **11.9 ± 6.2 ^2, 4^	22.8 ± 5.4 ^1, 3^	* 0.001
**20:5§EPA**					
Before	0.3 ± 0.6	0.4 ± 0.6	0.3 ± 05	0.3 ± 0.2	0.446
After	**† **4.9 ± 1.9 ^2, 4^	0.3 ± 0.2 ^1, 3^	**† **4.4 ± 0.4 ^2, 4^	0.4 ± 0.2 ^1, 3^	*0.001
**22:6§DHA**					
Before	1.9 ± 1.8	2.1 ± 1.4	2.0 ± 1.3	2.2 ± 1.2	0.331
After	**† **4.9 ± 2.8 ^2, 4^	2.0 ± 1.1 ^1, 3^	5.1 ± 1.4 ^2, 4 ^**†**	2.1 ± 1.3 ^1, 3^	*0.001

E+S = Exercise + Supplement; E = Exercise; S = Supplement; Con = Control; LA = Linoleic acid; AA = Arachidonic acid; EPA = Eicosapentaenoic acid; DHA = Docosahexaenoic acid,

**§ **Ratios represent number of carbon-carbon double bounds. Superscripts denote significantly differences among the groups (E+S = 1; E = 2; S = 3; and Con = 4).

**†**P < 0.05, significantly different from baseline values (within groups, baseline *vs*. week 24).

Values are mean ± SD

## Discussion

Prior evidence [2,12-32] indicates that both dietary fats and physical activity can influence bone health and alter inflammation. This study examined the combined effect of N-3 PUFA supplementation and exercise on BMD and inflammatory markers. The novel findings of this study are: 1) 24 wks of aerobic exercise in combination with N-3 PUFA supplementation synergistically increases L_2_-L_4 _and femoral neck BMD in healthy post-menopausal women, 2) inflammatory markers (TNF-α, IL-6) were attenuated with N-3 PUFA supplementation alone and exercise+supplementation, and 3) augmentation of estrogen, osteocalcin and 1, 25 Vit D levels were seen after 24 wk with exercise+supplementation only. These findings clearly show that the combination of PUFA supplementation with aerobic exercise provides numerous benefits on bone density and inflammation over exercise alone or supplementation alone. Additionally this study demonstrated that the reductions in inflammatory markers were related to the increases observed in BMD.

Although changes in bone turnover are slow, this study demonstrated that E+S resulted in a 15% increase in BMD in the L_2_-L_4 _region and a 19% increase in the femoral neck. Slight but non-significant increases in BMD were seen with exercise alone and with supplementation alone, but the N-3 PUFA acted synergistically with the exercise to result in profound changes in BMD in a 24 week period in this cohort of post-menopausal women.

Numerous factors may have contributed to these changes in BMD. All groups except the Con group showed increased CT and osteocalcin levels after the intervention period; although these increases were most dramatic in the E+S group. These findings are similar to those reported by Alev *et al*. [27] who found a 54% increase in CT levels after an aquatic exercise program, while another study reported 17.1 and 18.3 pg.ml^-1 ^increases respectively after endurance and strength exercise program than baseline (1.2 pg.ml^-1^) values [38]. Likewise previous research has also shown increases in osteocalcin with 12 and 16 weeks of exercise training [22]. These increases in osteocalcin concentrations have been observed with exercise training in both eumenorrheic, pre-menopausal [23] and post-menopausal women [24]. The underlying mechanisms of how exercise training and N-3 PUFA augments CT and osteocalcin levels are unknown, but these increases in CT and osteocalcin levels seem to be involved in increasing osteoblast-mediated bone formation in physiological concentrations and contribute to a more positive bone balance [12].

In addition to the enhancement of bone formation, our data has shows a decrease in bone resorption as reflected by a decrease in serum CTX levels. At 24 wks of the intervention CTX levels had decreased 32% in the E+S group, and this response seems to be a synergistic effect of the exercise and N-3 PUFA supplementation as there were no significant changes in either of these groups. Additionally, the changes in CTX coincide with the increases in L_2_-L_4 _and femoral neck BMD. Previous research has shown that N-3 PUFAs protect against age-related bone loss. Amy *et al*. [15] reported that incorporating walnuts and flaxseed into the diet to increase α-Linolenic Acid (ALA), and consequently decrease the N-6/N-3 ratio, reduced serum N-telopeptides (NTx) (a bone resorption marker) and maintained levels of serum bone-specific alkaline phosphatase (BSAP). Likewise animal studies have also shown an anti-resorptive effect of exercise [39,40]. James *et al*. [28] demonstrated that long-term moderate intensity resistance training in women on hormone therapy, reversed bone loss, decreased bone turnover, increased femur BMD, and caused a 9% decrease in CTX. Our findings suggest that the positive effects of exercise and N-3 PUFA intervention on bone mass in post-menopausal women may result from the anti-resorptive properties of either N-3 or exercise training induced by decreasing levels of serum CTX.

Our findings parallel previous reports that endurance exercise training increases serum 1, 25 Vit D [26,41] and decreases serum PTH concentrations [41]. With regard to the effect of exercise on calciotropic hormones, it is accepted that exercise promotes a positive calcium balance and increases skeletal mass, chiefly as a result of increased 1, 25 Vit D levels, decreased serum PTH and enhancement of intestinal calcium absorption [41].

Additionally research with N-3 PUFAs has demonstrated a protective effect of long chain N-3 PUFAs on age-related bone loss due to modulation of local factors and hormones such as insulin like growth factor I (IGF-1), PTH, and 1, 25 Vit D [17]. Our recently published data [12] also demonstrated that N-3 PUFA with and without weight-bearing exercise training in post-menopausal women increased CT and decreased PTH concentrations, indicators of bone formation and resorption, respectively. These hormonal changes were greater when supplementation was accompanied by exercise training. Thus the combined stimulus of aerobic exercise with N-3 PUFA consumption enhanced bone formation and suppressed bone resorption, resulting in an increased demand for minerals that was satisfied by an increase in serum 1, 25 Vit D and increased intestinal absorption of Ca^2+^. Possibly the increased Ca^2+ ^absorption suppressed serum PTH levels, and the decreased level of serum PTH in the E+S group may be related to increased concentration of EPA and DHA in membrane phospholipids as well as increased Ca^2+ ^absorption following N-3 PUFA consumption and participation in long-term aerobic exercise training.

Bone mineral density has been linked to estrogen levels, and the estrogen deficiency that occurs with menopause is tightly linked to low BMD. While exercise and supplementation alone had no significant effect on estrogen levels, we observed a 126% increase in estrogen levels in the E+S group. This agrees with our recent findings that found increased estrogen levels in post-menopausal women following 16 weeks weight-bearing exercise either alone or with N-3 PUFA supplementation [12], but is in contrast with the findings of McTiernan *et al*. [42] who reported reduced estrogen levels following a 12-month moderate-intensity exercise intervention in post-menopausal women. Most likely, the increased levels of estrogen in the present study were affected by coordinated changes in increased CT and decreased PTH levels that interplayed to allow the elevations in estrogen levels. According to previous investigations [43], when estrogen is withdrawn, such as after menopause, the rate of bone resorption is increased. Estrogen's major effect on bone tissue, therefore, may be to inhibit bone resorption rather than to promote bone formation. This anti-resorptive effect is mediated by the estrogen-induced synthesis and release of paracrine factors from osteoblast cells, which then control osteoclastic activity [43]. Estrogen increases the release of transforming growth factor-b (TGF-b) from osteoblasts, which subsequently inhibits activity of osteoclasts [44]. IL-6, a cytokine that increases osteoclastic activity, is regulated by estrogen, as IL-6 release from osteoblasts has been shown to be inhibited by estrogen. Further evidence of the anti-resorptive effect of estrogen is provided by observations of increased synthesis of IL-6 and increased osteoclastic cell number in estrogen-deficient women [45]. Estrogen not only affects bone metabolism at the cellular level but also has indirect effects systemically. Estrogen interacts with many other hormones, including CT and PGs, which result in a response of bone tissue. When the body is in a state of hypercalcemia, the C-cells are stimulated to release CT [45], which inhibits bone resorption. CT seems to be influenced by estrogen, as higher levels of estrogen have been strongly correlated with CT secretory capacity [46]. There is little known about the interaction between estrogen and PTH. Although estrogen does not affect PTH secretion, estrogen does seem to have an indirect effect on PTH action on bone. It has been suggested that estrogen inhibits bone cell responsiveness to PTH, thus reducing bone resorption [45]. This indirect effect further explains why estrogen withdrawal results in increased bone resorption.

The immunosuppressive effects of the either moderate intensity exercise training [47] or N-3 PUFAs [48-51] have been reported previously. Recently we showed that ingestion of N-3 PUFAs can be effective in ameliorating, eccentric exercise-induced, inflammatory markers including IL-6, PGE_2 _and TNF-α [51]. Previous studies [52] also revealed that reducing the consumption of fat calories from 36% to 27% with low fish-derived N-3 PUFA resulted in increased levels of IL-1β, and TNF-α production by peripheral blood mononuclear (PBMN) cells stimulated with lipopolysaccharide (LPS) while the same low fat diet with high fish-derived N-3 PUFA led to lowered amounts of these cytokines produced. Thus, decreased levels of IL-6, TNF-α and PGE_2 _in the present study can be explained by immunosuppressive effects of the either moderate intensity aerobic exercise training or N-3 PUFA. On the basis of previous studies, IL-6 possesses both pro-and anti-inflammatory actions [53]. The overproduction of pro-inflammatory IL-6 has been associated with multiple organ-system dysfunction and mortality. In contrast, immunoregulatory function of anti-inflammatory IL-6 inhibits the production of several pro-inflammatory cytokines including IL-6 and TNF-α by altering the accessory cell function of macrophages [54]. Generally following exercise, pro-inflammatory cytokine levels are counterbalanced by anti-inflammatory cytokine levels promoting homeostasis; however if levels are unrestrained, post exercise infection may occur [55]. Hence it is possible that the decreased levels of IL-6 (~40%) and TNF-α (~80%) observed with the E+S and S interventions may be related to immunoregulatory function of anti-inflammatory IL-6. Likewise, since PTH regulates the circulating levels of the inflammatory cytokines IL-6 and TNF-α [56], we reasoned that in the present study aerobic exercise and N-3 PUFA consumption, by suppressing PTH production, might also decrease the serum IL-6 and TNF-α levels.

Paralleling the postmenopausal bone loss, increased production of cytokines is a possible mechanism that contributes to post-menopausal bone loss [57]. Cytokines play both a direct and an indirect role in the regulation of osteoclast and osteoblast activities [58]; therefore, a decrease in cytokine production may be beneficial to BMD. Consistent with our findings, a recent study has suggested that bone loss in post-menopausal women is mediated by increased production of cytokines such as IL-1, IL-6, IL-8 and TNF-α [59], and our data support this. Inflammatory processes can up-regulate many cytokines, such as IL-1, IL-6 and TNF-α, which strongly stimulate CRP production from the liver [60,61] as well as inducing bone resorption [7,9,62]; and increased bone resorption may result in increased bone turnover and decreased BMD. In support of this hypothesis, the production of IL-1, IL-6 and/or TNF-α by peripheral blood monocytes was positively correlated with bone resorption or spinal bone loss in healthy pre- [63] and post-menopausal [64] women, and serum IL-6 concentrations predicted femoral bone loss in healthy post-menopausal women [65]. Also, increased levels of PGs, especially PGE_2 _(known to be potent activators of bone remodeling) are found in several disorders characterized by chronic inflammation [66].

Data from supplement studies in the elderly indicate that N-3 PUFA intakes may promote bone mineral maintenance in this population [67]. Eighteen-month of EPA and γ-linolenic acid supplementation in elderly post-menopausal women revealed that BMD at the lumbar spine was maintained and BMD of the femoral neck was increased [67]. In a cohort of growing rats fed on a semisynthetic diet supplemented with tuna oil, Kruger and Schollum [68] found that DHA concentrations in red blood cell membranes were associated with BMD, and with calcium absorption in bone. Likewise, Weiler and Fitzpatrick-Wong [69] found that higher concentrations of plasma DHA were associated with an attenuated bone resorption in piglets fed on diets containing different ratios of N-6 to N-3 fatty acids. An increased consumption of N-3 PUFA decreases the AA:EPA ratio, PGE_2 _concentration, and release from bone. This cascade of effects is potentially important, as PGE_2 _has been reported to stimulate bone resorption [70]. EPA serves as a precursor for the formation of PGE_3_, which also can stimulate bone resorption. However, the conversion of EPA to PGE_3 _is much less efficient than is the conversion of AA to PGE_2 _[71]. Although measurements of bone PGs were not taken in the present study, the neutrophil phospholipids PUFA content results showed that EPA and DHA in the cell membranes of neutrophils were significantly greater in the E+S (4.9% and 4.4%, respectively) and S (4.9% and 5.1%, respectively) groups than in either the E or the Con groups, while AA and LA were significantly reduced. It is possible that this shift in fatty acid synthesis decreased production of PGE_2 _within the bone while having minimal effect on PGE_3 _formation. Therefore, a decrease in cytokines and PGE_2 _production by long-term aerobic exercise and cyclooxygenase (COX)-2 inhibitor N-3 PUFAs in the present investigation can be attributed to increased bone health measured by L_2_-L_4 _and femoral neck BMD.

Our results also support the hypothesis that the elevated L_2_-L_4 _and femoral neck BMD, estrogen, osteocalcin, and CT experienced during aerobic exercise and N-3 PUFA consumption in healthy post-menopausal women are negatively associated with the serum inflammatory markers TNF-α and PGE_2_. Likewise, elevated CT and decreased PTH levels during the study period are associated (negatively and positively, respectively) with serum IL-6. These changes in inflammatory indices following long-term aerobic exercise training and N-3 PUFA supplementation may contribute significantly to the inverse relationship between inflammatory markers and osteoporosis. Together these findings allows the speculation that increased L_2_-L_4 _and femoral neck BMD measures following exercise training and N-3 PUFA consumption may be related to increased levels of estrogen, osteocalcin, and CT as well as decreased PTH, IL-6, TNF-α and PGE_2 _levels in post-menopausal women. In rodent models, IL-6 mediated stimulation of osteoclast activity and regulation in the presence of estrogen depletion has been well reported [72,73]. In the present study, lack of association between serum IL-6 and other variables (with the exception of CT and PTH) following exercise and N-3 PUFA intervention can be attributed to the elevated estrogen levels to some extent.

In conclusion, aerobic exercise training plus N-3 PUFA supplementation was effective in reducing chronic inflammation and increasing BMD in postmenopausal women. These changes in inflammatory markers are related to indices that favour enhancement of BMD in healthy sedentary post-menopausal women. Further studies on the physiologic effects of N-3 PUFAs and exercise training on bone metabolism and bone quality to prevent or treat osteoporosis are needed.

## Abbreviations

N-3: Omega-3; BMD: Bone mineral density; E+S: Exercise + supplement group; E: Exercise group; S: Supplement group; Con: Control group; L: Lumbar spine; TNF: Tumor necrosis factor; IL: Interleukin; PG: Prostaglandin; 1, 25 Vit D: 1, 25-dihydroxyvitamin D_3_; CTX: C-telopeptide; PTH: Parathyroid hormone; CT: Calcitonin; PUFAs: Polyunsaturated fatty acids; EPA: Eicosapentaenoic acid; CRP: C-reactive protein; DHA: Docosahexaenoic acid; ELISA: Enzyme-linked immunosorbent assay; RIA: Radioimmunoassay; ANOVA: Analysis of variance; LA: Linoleic acid; AA: Arachidonic acid; ALA: α-Linolenic Acid; NTx: N-telopeptides; BSAP: Bone-specific alkaline phosphatase; IGF-1: Insulin like growing factor I; TGF-b: Transforming growth factor-b; PBMN: Peripheral blood mononuclear cells; LPS: Lipopolysaccharide; GLA: γ-linolenic acid; COX: Cyclooxygenase; BMI: Body mass index; VO_2max_: Maximal oxygen uptake.

## Competing interests

The authors declare that they have no competing interests.

## Authors' contributions

BT, JK and BHM had substantial contributions to conception and design, acquisition, analysis, and interpretation of data. BHM was responsible for drafting the article, revising it critically for important intellectual content, and final approval of the version to be published. JK and KS revised manuscript for grammar and literature. All authors read and approved the final manuscript.
